# Chronic arthritis in children and adolescents in two Indian health service user populations

**DOI:** 10.1186/1471-2474-5-30

**Published:** 2004-08-27

**Authors:** Joyce Mauldin, H Dan Cameron, Diane Jeanotte, Glenn Solomon, James N Jarvis

**Affiliations:** 1 Dept. of Pediatrics, University of Oklahoma College of Medicine, BSEB #235A, Oklahoma City, OK, 73104 USA; 2 Oklahoma City Area Indian Health Service, Five Corporate Plaza, 3625 NW 56th Street Oklahoma City, OK, 73112 USA; 3 Billings Area Indian Health Service, 2900 4th Avenue North, Billings, MT 59101 USA; 4 Current address: Oklahoma City Area Indian Health Service, Five Corporate Plaza, 3625 NW 56th Street, Oklahoma City, OK, 73112 USA; 5 Current address: Arthritis & Immunology Program, Oklahoma Medical Research Foundation, Oklahoma City, OK, 73104 USA

## Abstract

**Background:**

High prevalence rates for rheumatoid arthritis, spondyloarthopathies, and systemic lupus erythematosus have been described in American Indian and Alaskan Native adults. The impact of these diseases on American Indian children has not been investigated.

**Methods:**

We used International Classification of Diseases-9 (ICD-9) codes to search two Indian Health Service (IHS) patient registration databases over the years 1998–2000, searching for individuals 19 years of age or younger with specific ICD-9-specified diagnoses. Crude estimates for disease prevalence were made based on the number of individuals identified with these diagnoses within the database.

**Results:**

Rheumatoid arthritis (RA) / juvenile rheumatoid arthritis (JRA) was the most frequent diagnosis given. The prevalence rate for JRA in the Oklahoma City Area was estimated as 53 per 100,000 individuals at risk, while in the Billings Area, the estimated prevalence was nearly twice that, at 115 per 100,000. These rates are considerably higher than those reported in the most recent European studies.

**Conclusion:**

Chronic arthritis in childhood represents an important, though unrecognized, chronic health challenge within the American Indian population living in the United States.

## Background

As a group, the rheumatic diseases of childhood represent one of the most common chronic disease conditions in children [1]. These illnesses have global distribution [2-5], but little information exists regarding either prevalence or phenotypic expression of these diseases in children in any population other than North American and European whites [6]. Aggarwal and colleagues [7] have reported their experience with juvenile rheumatoid arthritis (JRA) on the Indian subcontinent, and their findings suggest that the patterns of disease reported in Europe and North America are not seen in that population. Most conspicuous in Aggarwal's study was the relative rarity of the pauciarticular form of JRA, in contrast to European and North American populations, where that subtype accounts for 50 to 75% of the cases [3,8-11]. The relative rarity of pauciarticular JRA in non-European populations has been documented in children of Kuwait [5], Turkey [12], Thailand [13], Japan [14] and South Africa [15] as well as in African American children in Detroit [16]. Further evidence that examining prevalence rates of rheumatic diseases in specific populations may be informative comes from studies of systemic lupus erythematosus (SLE). Studies from Great Britain, for example, indicate that the prevalence rates for SLE in people of Afro-Caribbean descent may be 4–8 times higher than that in Caucasians [17,18].

Prevalence rates of rheumatic disease in North American Indian/First Nations populations have been reported in small studies from single tribes. From these studies, significantly higher prevalence rates for rheumatoid arthritis (RA) have been reported in adults from tribes living in the Great Lakes region [19], the Pacific Northwest [20], the Southwest [21] and Canada [22-24].

To our knowledge, a comprehensive survey of rheumatic diseases affecting children, adolescents, and young adults has not been reported in any non-Caucasian population. Because both our clinical experience here in Oklahoma suggests that rheumatic diseases in children may also be more prevalent in the American Indian population compared with Caucasians, we undertook a search of the Oklahoma City Indian Health Service (IHS) user population databases in order to develop prevalence estimates of rheumatic diseases in American Indian children and adolescents. We performed the same queries from the database in the Billings Area IHS office as a basis of comparison.

## Methods

### Populations in the billings and Oklahoma City areas

The Oklahoma City Area IHS serves a population of 291,288 individuals, most of whom reside in Oklahoma, with small numbers living in the neighboring states of Kansas and Texas [25]. IHS services are limited to members of federally recognized tribes, 39 of whom have tribal headquarters in Oklahoma. The 39 federally recognized tribes [26] represent people from multiple Native cultures including Eastern Woodlands tribes (e.g., Cherokee, Delaware, Seneca), Southeastern tribes (e.g., Creek, Choctaw, Chickasaw, Seminole), Southwestern tribes (e.g., Apache), as well as tribes who have long been resident on the southern Great Plains (e.g., Kiowa, Comanche, Southern Cheyenne). Tribal membership is determined by the tribes themselves and may or may not include specific blood quantum requirements for membership. Historical factors, the absence of reservations, and the proximity of European-descended people in Oklahoma has resulted in significant admixture between Native and Caucasian populations in many parts of the Oklahoma City Service Area.

The Billings Area IHS serves a population of 72,591 individuals. The tribes in this service area, a significant proportion of whom live on 8 reservations located in Montana and Wyoming, consist largely of northern plains tribes (e.g., Crow, Sioux, Blackfeet).

In both Areas, the population is younger than the population of the United States as a whole, with 40 percent of the population 19 years of age or younger [27].

### Database search

International Classification of Diseases, Ninth Revision, Clinical Modification (ICD-9) codes were used to search the Oklahoma City and Billings Area IHS National Patient Information Reporting Systems and Patient Registration user databases [28] over a three-year period (1998–2000) to identify individuals with rheumatic diseases.

Outpatient data over this three-year period was gathered for the user population ≤ 19 years of age and included: patient chart number (with the chart number scrambled to protect patient identity), date of birth, sex, date of visit and diagnosis (the IHS database allows at least 9 diagnoses to be recorded on any given patient). The codes used and the diagnoses denoted by those codes are listed in Table 1. Data were downloaded into a standard database (Microsoft Excel), which was then searched to eliminate duplicate records and to sort patients for each year on the basis of age, sex, and diagnosis.

A case was defined as any person who was 19 years of age or younger on January 1,1998, January 1, 1999 and January 1, 2000 and whose diagnoses included at least one of the entities listed in Table 1.

The population at risk was defined as the number of individuals ≤19 years of age within the IHS user population (i.e., eligible individuals who have used the IHS facilities at least one time in three years) [29]. The user population (i.e., people who actually used IHS services) may differ from the IHS *service *population, which includes all individuals who are eligible to receive IHS services. Estimation of disease prevalence was based on three assumptions: (1) that the diagnoses recorded were, in fact, accurate; (2) that the population at risk did not change significantly over the three-year period; (3) that the three-year mortality rate for the diseases of interest was no greater in the IHS user population than for all races in the United States.

For the Oklahoma City Area, accuracy of the IHS database information was assessed by matching the IHS identification number of known JRA cases followed at the Children's Hospital of Oklahoma with the same number in the IHS patient databank to be sure that known patients were identified and coded accurately.

## Results

### Rheumatoid arthritis/juvenile rheumatoid arthritis

**Figure 1:**
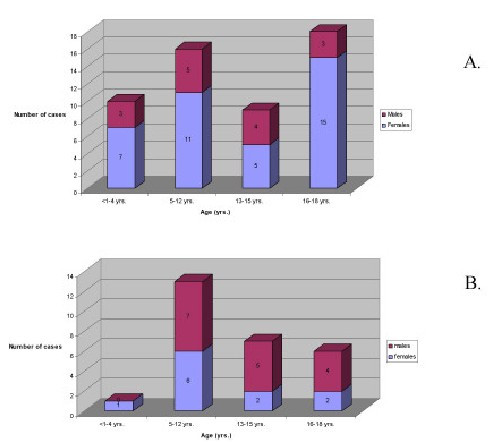
Bar graphs showing the age and sex distributions of children with JRA identified in the Oklahoma City Area (A) and Billings Area (B) IHS databases. In each Area, there was a conspicuous under-representation of both pre-school children and early adolescents diagnosed with JRA.

Rheumatoid arthritis and juvenile rheumatoid arthritis (RA/JRA; #ICD-9 # 714.0, 714.30) were the most frequent rheumatic disease diagnoses recorded in individuals ≤ 19 years of age in both IHS areas. In the Oklahoma City Area, we identified 62 individuals (45 females and 17 males) with these diagnoses. Assuming a population at risk of 117,409 (i.e., individuals 19 years of age or younger; source: IHS Headquarters office, data processing services unit, Albuquerque, NM), this gives a crude prevalence rate of 53 cases per 100,000 at risk. The prevalence rate for Billings was considerably higher. The 714.0 and 714.3 codes identified 33 individuals in the Billings Area. Based on a population at risk of 28,724, the prevalence estimate for Billings was 115 per 100,000 at risk (see Table 2). The age distribution of affected children, adolescents, and young adults in both areas differed from what has been reported in studies from predominantly European or European-descended populations (Figures 1A and 1B). There is a distinct biphasic distribution of JRA prevalence by age in Caucasians, with peaks in the late preschool years and in early adolescence [30,31]. Data from both IHS databases show a distinct peak at age 5–12 years with proportionately smaller numbers of patients in the preschool and early adolescent age groups. In both Areas, peaks in late adolescence and early adulthood are observed, consistent with our observation that rheumatoid arthritis is a disease of young adults in this population (Mauldin *et al*, manuscript in preparation). The absence of a prevalence peak in the preschool years may reflect the almost complete absence of children with monoarticular or pauciarticular JRA in the IHS user population, consistent with previous studies in non-European populations [5,7,8,13,15,32,33]. We found no individuals in the database with the ICD-9 code commonly used to denote pauciarticular-onset JRA (#ICD-9 # 714.32). In the Oklahoma City Area we found a single child (a 12 year old female) diagnosed with monoarthritis (#ICD-9 # 714.33).

Included in the above analyses are individuals who may not fit established criteria for a diagnosis of JRA. Since we did not examine age at onset, it is impossible to know whether a 19 year old identified as having rheumatoid disease was age 7 or age 17 at disease onset. JRA diagnosis criteria stipulate that patients must have disease onset at 15 years of age or younger [34]. When data were analyzed to include only children 15 years of age or younger, we identified 35 patients (23 females, 12 males) in the Oklahoma City Area and 21 patients in the Billings Area (9 females, 12 males) with a diagnosis of JRA. Based on the populations at risk of 87,936 (Oklahoma City) and 21,777 (Billings) this yields an estimated prevalence rate of 40 per 100,000 in the Oklahoma City Area and 96 per 100,000 in the Billings Area. Both of these estimates are more than twice the prevalence derived from a recent European study (14.8 per 100,000) [35].

In order to test the integrity of the IHS database in the Oklahoma City Area, we matched known cases of JRA followed at the Children' Hospital of Oklahoma (CHO; n = 15) by IHS identification number with patients in the database. All 15 of the children followed at CHO were identified within the database and correctly identified by subtype.

We did not have access to patient records in the Billings Area, but do have access to databases at other IHS facilities. At a large facility in the Aberdeen Area (comprising the states of North Dakota, South Dakota, Nebraska, and Iowa and serving a patient population very similar to that served in the Billings Area) we identified 20 children with JRA using the same search strategy as that used in for the Billings and Oklahoma City Areas. Subsequent chart review demonstrated that 17 of these children had strong clinical evidence to confirm the diagnosis of JRA, while diagnosis could not be supported or excluded in the other three. The estimated prevalence for JRA in the population served by this facility calculates to 236/100,000, within the same order of magnitude by considerably higher than the Billings Area estimate.

### Spondyloarthropathy

The second most common diagnoses identified in each Area database were the group of illnesses collectively denoted spondyloarthropathies (#ICD-9 # 720.0, 720.1, 720.2, 720.8, 720.89). Although these illnesses are sometimes viewed as distinct entities, they share sufficient common features that allow them to be grouped for purposes of this analysis. These common features include: (1) male sex preponderance; (2) arthritic involvement of the axial skeleton (e.g., sacroiliac joints); (3) extra-articular musculoskeletal involvement (e.g., bursitis, enthesitis); and (4) extra-articular (e.g., ocular, genito-urinary) inflammation. In both white [36-38] and American Indian patients [39-43], the human class I histocompatibility complex antigen HLA-B27 constitutes a strong risk factor [44-47].

We identified 20 patients (12 females, 8 males) with spondyloarthropathy in the Oklahoma City Area IHS database, giving an overall crude prevalence rate of 17 per 100,000 at risk. Included in this group are three patients (all female) with psoriatic arthritis (ICD-9 Code # 696.0). In the Billings Area, we identified 12 individuals (7 females and 5 males) with ICD-9 codes used to identify patients with spondyloarthopathy, yielding a prevalence rate of 42 per 100,000 at risk. These included one patient (a 12 year old male) with ankylosing spondylitis (#ICD-9 # 720.0), one (a 5 year old male) with psoriatic arthritis (#ICD-9 #696.0), and 10 patients (6 females and 4 males) with nonspecific sacroiliitis (#ICD-9 # 720.2). The Oklahoma and Billings Area estimates were within the range previously reported for childhood spondyloarthropathies (e.g. ankylosing spondylitis) in the United States and United Kingdom (12 to 33 per 100,000) and Mexico (13 to 65 per 100,000) [48], but slightly lower than previously estimated prevalence rates of 29 per 100,000 (all spondyloarthropathies) for First Nations children in western Canada [22]. The female-to-male preponderance in both Areas was unusual and has not been, to our knowledge, reported with any previous population.

## Discussion

Although rare individually, the rheumatic diseases, taken together, are among the most common chronic health conditions affecting children [1]. Exact prevalence rates among children living in the United States are difficult to obtain, owing, in large part, to the de-centralized delivery of health care in this country. The IHS represents an exception to that decentralization and is, arguably, the closest representation to a nationalized health care delivery system currently functioning within the United States. Thus, records and data available through the IHS represent a unique opportunity to assess population-wide health needs not otherwise available to child health researchers in this country. This report provides a first-ever population-wide estimate of the prevalence of chronic arthritis in American Indian children living within the United States. While the prevalence rate for the Oklahoma City Area was within the same order of magnitude as the most recent reports from Europe [35], the prevalence rate in the Billings Area was nearly 10 times this recent European estimate. These findings are consistent with earlier studies of rheumatoid disease in American Indian adults, where prevalence rates 10 times higher than the general population were reported [49,50]. It should be pointed out, however, that prevalence estimates of JRA vary widely, ranging between 16 to 113 per 100,000 [30,31,51-55].

The reasons for the discrepancy in prevalence estimates between the Oklahoma City and Billings Areas are not clear. One possibility is that the northern plains tribes are particularly susceptible to rheumatoid disease in ways that other groups (e.g., Eastern Woodlands or Southwestern tribes) are not. It is also possible that the difference in ethnic composition of the two populations accounts for this difference. While many Oklahoma tribes require at least a 25% blood quantum of tribal ancestry (e.g., the Kiowa tribe [56]), other tribes require only proof of descent from an individual on the original Dawes rolls of 1893 [57]. Thus, the Oklahoma City Area includes many individuals whose degree of American Indian ancestry is 1/4 or less and may include individuals with less than 1/64 American Indian ancestry. In contrast, there has been less intermingling between Caucasian and American Indian populations on the northern plains, and a larger percentage of the Billings Area population includes individuals with full-blooded American Indian ancestry.

Our study once again points out the rarity of the pauciarticular form of JRA in non-European populations. In studies of European and European-descended populations, pauciarticular JRA is the most common form of chronic childhood arthritis [3,8].

The Oklahoma City Area database listed a single child with ICD-9 codes #714.32 (pauciarticular JRA) or 714.33 (monoarticular arthritis), the codes used to identify such children. These findings are consistent with reports from the Indian subcontinent,[7] Kuwait [5], Turkey [12], Thailand [13], Japan [14], South Africa [15], and with our experience with African American children in Detroit [16].

The slight female-to-male preponderance for spondyloarthopathy is also worth noting. High prevalence rates for spondyloarthopathies have been noted in both Northwestern and Southwestern tribes [24,41-44]. However, in these studies, a strong male preponderance was noted. Whether the findings from Oklahoma City and Billings represent a novel finding or inaccuracies in the ICD-9 coding await confirmatory studies, as we discuss below.

An important limitation to this study is the fact that we did not have the means to verify every individual case listed in Oklahoma City database and were unable to confirm any diagnosis in the Billings Area database. However, our limited test of the accuracy of the Oklahoma City data provided surprising confirmation of the accuracy of coding for known cases. While we could not confirm any of the Billings cases, our search of the database of a single IHS facility in the neighboring Aberdeen Area corroborated the prevalence statistics we derived from the Oklahoma City and Aberdeen databases. Indeed, our experience suggests that a search strategy like the one we used is likely to under-estimate rather than over-estimate the prevalence of rheumatic disease in the IHS user population.

We are aware that there are many factors that might overestimate disease prevalence using this type of database search. The first is the possibility that a given ICD-9 code might have been used to designate a "working" diagnosis that was never established by the patient's clinical course. The second opportunity for overestimation of prevalence would occur if children were systematically misdiagnosed. This could occur easily if physicians use serologic data as the sole criterion for diagnosis. For example, many physicians routinely screen children with musculoskeletal complaints using antinuclear antibody (ANA) tests. However, the prevalence of low-titer positive ANA tests is extraordinarily high in the pediatric population [58]. Thus, if ANA-positive children with musculoskeletal pain [59] are listed as having "JRA," then there would be a gross overestimation of the actual prevalence.

Similarly, there are factors that might have led to underestimation of JRA prevalence by relying solely on a three-year database search. Children or adolescents with well-controlled JRA may not have seen an IHS physician during the relevant time period, and thus would have been excluded. Similarly, physicians who rely on rheumatoid factor tests as a diagnostic criterion for JRA might fail to diagnose the disease in a child, since only a small number of children with JRA have detectable IgM rheumatoid factor [60].

The ideal method for obtaining true disease prevalence rates would include rigorous, pro-active case finding in a known population at risk. This approach was taken by Manners and Diepeveen in a study of school children in Australia [61]. Using such an approach, these authors reported a prevalence rate of 4 per 1,000 for JRA, significantly higher than any previous estimates. We are now preparing a similar project involving American Indian communities on the northern plains.

## Conclusion

We conclude that the rheumatic diseases of childhood may represent a significant burden of morbidity in these two IHS user populations. More detailed studies with rigorous case ascertainment are required to follow up these preliminary data.

## Competing Interests

None declared.

## Authors' Contributions

Dan Cameron provided data from the IHS database in Oklahoma City, and Diane Jeannotte provided the Billings Area data. Joyce Mauldin and Glenn Solomon performed the database searches. Dr. Jarvis directed this study and assisted in data analysis and interpretation.

## Pre-publication history

The pre-publication history for this paper can be accessed here:



## Figures and Tables

**Table 1:** 

ICD-9 CODES	Disease
696.0	Psoriatic arthropathy
695.4	Lupus erythematosus (discoid)
710.0	Systemic lupus erythematosus
710.1	Systemic sclerosis
710.2	Sicca syndrome
710.3	Dermatomyositis
710.4	Polymyositis
710.8	Other specified diffuse diseases of connective tissue
710.9	Unspecified connective tissue disease
714.0	Rheumatoid arthritis
	Other rheumatoid arthritis of visceral or systemic involvement
714.2	Juvenile chronic polyarthritis
714.30	Polyarticular juvenile rheumatoid arthritis, chronic or unspecified
714.33	Monoarticular juvenile rheumatoid arthritis
Spondyloarthropathies	
720.0	Ankylosing spondylitis
720.1	Spinal enthesopathy
720.2	Sacroilitis, not elsewhere classified
720.8	Other inflammatory spondyloarthopathies
720.89	Other spondyloarthropathies

**Table 2:** Rheumatic Diseases in Children and Adolescents Identified in the Oklahoma City and Billings Area Databases

**Area**	**Oklahoma City Area**		**Billings**	
**Disease Entity**	**Number**	*Prevalence ***(Estimated)**	*Number*	*Prevalence ***(Estimated)**

*RA/JRA*	62	53/100,000	33	115/100,000
*Spondyloarthopathy*	20	17/100,000	12	42/100,000
*SLE*	13	11/100,000	4	14/100,000
*PMS/DMS*	5	4/100,000	4	13/100,000
*Other*	8		2	
